# The application of genetic testing technology in kidney transplantation: precision matching, non-invasive monitoring and personalized management

**DOI:** 10.3389/fimmu.2025.1713293

**Published:** 2026-01-02

**Authors:** Yalong Zhang, Hao Wang, Rui Yan, Kangyu Wang, Jiangwei Man, Li Yang

**Affiliations:** 1 Department of Urology, The Second Hospital and Clinical Medical School, Lanzhou University, Lanzhou, Gansu, China; 2 Gansu Province Clinical Research Center for Urinary System Disease, Lanzhou, Gansu, China

**Keywords:** kidney transplantation, genetic testing, donor-derived cell-free DNA, pharmacogenomics, HLA typing, rejection monitoring, precision medicine, infection risk prediction

## Abstract

Kidney transplantation remains the treatment of choice for patients with end-stage renal disease, yet its long-term success continues to face major challenges, including organ shortage, rejection, and drug toxicity. With the advancement of genetic testing technologies, transplant management is progressively shifting from empirical practice toward precision medicine. This review systematically outlines four core applications of genetic testing in kidney transplantation: from pre-transplant precision donor-recipient matching and risk stratification, to peri-operative pharmacogenomics-guided immunosuppression, and finally post-transplant noninvasive rejection monitoring and infection management. By integrating high-resolution HLA typing, epitope mismatch analysis, donor-derived cell-free DNA monitoring, urinary biomarker detection, genotyping of drug-metabolizing genes such as CYP3A5, and assessment of host susceptibility variants, genetic technologies have significantly improved transplant outcomes. Despite persistent challenges in standardization, clinical translation, and ethical considerations, emerging innovations including microfluidics, nanopore sequencing, and organoid modeling are expected to further accelerate the transition of kidney transplantation into a new era of comprehensive precision management.

## 1 Introduction

Kidney transplantation is the treatment of choice for patients with end-stage renal disease (ESRD), offering significant improvements in quality of life and long-term survival (1). However, its sustained success continues to face multiple challenges. Globally, the gap between organ supply and demand is widening, driven by an aging population and the growing prevalence of uremia, resulting in a steady increase in kidney transplant demand that far outpaces the growth in donor organ availability (2). To mitigate this imbalance, transplant programs have increasingly relied on marginal kidneys, including those from elderly donors, donors with comorbidities, or donation after cardiac death (3). While such strategies expand the donor pool, they also increase the risk of early graft dysfunction and compromise long-term outcomes (4). Advances in modern immunosuppressive regimens have markedly improved short-term transplant success, with one-year graft survival rates now exceeding 90% in most centers (5). Nevertheless, long-term outcomes remain suboptimal, with ten-year graft survival plateauing at approximately 50% (6). The major barriers to sustained graft function include chronic rejection, toxicities associated with long-term immunosuppression, and post-transplant infections, all of which remain central obstacles to improving long-term efficacy (7).

Conventional diagnostic and therapeutic approaches for kidney transplantation have significant limitations. Monitoring of serum creatinine and estimated glomerular filtration rate (eGFR) for early detection of rejection is hindered by delayed responsiveness and insufficient specificity, with abnormalities often emerging only after irreversible graft injury has already occurred (8). Although protocol biopsies are considered the gold standard for diagnosing rejection, their widespread use is limited by the invasive nature of the procedure, potential complications such as bleeding and infection, high medical costs, and sampling errors, which collectively reduce feasibility and patient compliance (9). Furthermore, immunosuppressive drug dosing in clinical practice is largely based on patient body weight and empirical experience. These agents have a narrow therapeutic window, and marked interindividual variability in pharmacokinetics and pharmacodynamics-ranging from 30% to 50%-makes it challenging to balance efficacy and toxicity (10). As a result, some patients develop nephrotoxicity or neurotoxicity due to supratherapeutic drug exposure, while others experience subtherapeutic concentrations that increase the risk of allograft rejection.

Against this background, genetic testing technologies are driving a paradigm shift in kidney transplantation from an experience-based approach to precision medicine. By analyzing donor and recipient genetic profiles, these techniques enable deeper genetic-level matching, noninvasive molecular monitoring of rejection, individualized immunosuppressive therapy guided by pharmacogenomics, and accurate prediction of infection risk. While previous reviews have largely focused on isolated technologies, such as the specific application of donor-derived cell-free DNA or pharmacogenomics in isolation, there is a lack of comprehensive literature integrating these diverse genetic tools into a unified clinical workflow. This review distinguishes itself by systematically outlining the application of genetic testing across the entire clinical timeline of kidney transplantation. We propose a structured precision management framework organized into three clinical phases. In the pre-transplantation phase, the focus is on precision donor-recipient matching, including both human leukocyte antigen (HLA) and non-HLA genetics, alongside inherent risk stratification for factors such as infection susceptibility. During the peri-operative and maintenance phase, pharmacogenomics is utilized to guide the initiation and adjustment of immunosuppressive therapy. Finally, the post-transplantation surveillance phase employs noninvasive molecular monitoring tools, specifically donor-derived cell-free DNA and urinary biomarkers, for the early detection of rejection. By synthesizing biological foundations with clinical evidence, we aim to provide a roadmap for transitioning kidney transplantation from an empirical practice to a new era of comprehensive precision management. The clinical significance and typical decision points of genomics across the pre-, peri-, and post-transplantation phases are summarized in **Table 1**.

**Table 1:** The clinical significance and decision points of genomics.

Application Scenarios	genetic marker	Clinical significance	Typical decision points
Matching optimization (28–30, 33)	High-resolution HLA-DQ/DP typing	Reducing the risk of acute rejection and ABMR	Precise matching; developing desensitization and matching strategies for individuals with high PRA
Haplotype matching and susceptibility to rejection (37–39)	KIR-ligand mismatch	May increase NK cell-mediated rejection	Incorporated into the comprehensive risk assessment, supplemented by closer monitoring
Rejection of susceptibility (40–45)	Cytokines (TNF-α, IFN-γ)	Differences in the intensity of inflammatory responses	Combined with other indicators for individualized monitoring
Donor risk (50–52)	APOL1 high-risk genotype	Associated with graft functional decline	Donor assessment, follow-up strategy and ethical communication
Susceptible to infection (53–57)	TLR4 rs4986790	CMV/Bacterial Infection Susceptibility	Enhancing follow-up frequency and prevention strategies
Pharmacogenomics (58–64)	CYP3A5	Tacrolimus metabolism/dose requirements	*1 Carriers often require an increased initial dose; closed-loop TDM is employed.
Pharmacogenomics (70–73)	UGT1A9	Mycophenolic acid exposure/toxicity	Patients with rapid metabolism (*1 variant carriers) require optimization of dosage or treatment regimen.
Non-invasive rejection monitoring (76–88)	dd-cfDNA	High sensitivity/specificity for ABMR, early warning	Threshold-triggered biopsy/treatment assessment; dynamic follow-up
Non-invasive rejection monitoring (91–96)	Urinary mRNA (CXCL9, TIM-3)	Reflecting renal T-cell/immune activation	When combined with serum creatinine, it enhances negative predictive value and facilitates early identification.
Infection Management (104–108)	BK/CMV viral load qPCR	Postoperative infection monitoring	Preventive and Early Treatment Decisions

ABMR, Antibody-Mediated Rejection; PRA, Panel Reactive Antibody; dd-cfDNA, Donor-derived cell-free DNA; TDM, Therapeutic Drug Monitoring; APOL1, Apolipoprotein L1.

## 2 Pre-transplantation: precision matching and risk stratification

The pre-transplantation phase is the cornerstone of long-term success. Advances in genetic testing have enabled a shift from conventional serology to high-resolution molecular sequencing, facilitating precise matching of both HLA and non-HLA factors, as well as stratification of inherent genetic risks.

### 2.1 HLA genotyping and precision donor–recipient matching

The HLA system, which encodes the major histocompatibility complex (MHC), is a cluster of genes located on the short arm of chromosome 6 (11). Genetic polymorphisms within HLA molecules are the primary determinants that trigger transplant rejection (12).

#### 2.1.1 Evolution of HLA typing technologies

Early HLA typing was primarily performed using polymerase chain reaction with sequence-specific primers (PCR-SSP) or sequence-specific oligonucleotide probes (PCR-SSO). PCR-SSP employs primers designed for specific alleles to amplify target fragments. The method is relatively simple and rapid, producing results within a few hours, and is suitable for low-resolution, antigen-level screening (13). However, it has inherent limitations in resolution and throughput: it can identify allele groups or antigen-level markers such as HLA-A02 but cannot reliably distinguish highly polymorphic allele subtypes such as HLA-A02:01 versus HLA-A02:06. In addition, each assay covers only a limited number of loci, often requiring multiple parallel reactions. Similar to traditional serological techniques, PCR-SSP is prone to ambiguous typing results and insufficient resolution, necessitating confirmatory testing with higher-resolution methods (14). PCR-SSO, which relies on hybridization with sequence-specific probes, allows simultaneous detection of multiple alleles (15). Despite this advantage, the method is relatively labor-intensive, with complex workflows and interpretation. Nevertheless, its relatively low reagent cost and modest equipment requirements have sustained its continued use in some regional typing laboratories, particularly for routine screening.

With the advancement of molecular biology, Sanger sequencing technology was introduced into HLA typing, commonly referred to as sequence-based typing, or PCR-Sanger Sequencing-Based Typing (SBT). This technique sequences key exonic regions of HLA genes to directly obtain allele sequences, enabling high-resolution four-digit typing and substantially improving accuracy (16). For decades, it has been regarded as the gold standard in HLA typing. Nevertheless, the limitations of this method are also significant. First, sequencing is generally confined to specific regions such as key exons, which makes it prone to phase ambiguity in heterozygous samples, leading to uncertainty in allele combinations. Studies have shown that a considerable proportion of results from conventional Sanger sequencing-based typing remain ambiguous and require clarification through supplementary PCR-SSP/SSO assays or cloning-based sequencing (17). Second, the workflow is relatively time-consuming, with a turnaround time of approximately one to two days per sample. The cost is also high, often reaching several hundred US dollars per specimen, and throughput is limited, making the method unsuitable for large-scale rapid typing. Consequently, SBT is primarily employed as a confirmatory test in high-risk or clinically challenging cases (18).

The advent of high-throughput Next-Generation Sequencing (NGS) has brought a revolutionary breakthrough to HLA typing. NGS enables large-scale parallel sequencing of HLA genes, allowing full-length reads that encompass both exons and introns. This not only provides ultra-high-resolution allele typing but also facilitates the discovery of novel polymorphic sites (19). Compared with traditional methods, NGS offers distinct advantages in throughput, accuracy, and the depth of genetic information obtained. It enables simultaneous typing of multiple loci, including HLA-A, HLA-B, HLA-C, HLA-DR, HLA-DQ, and HLA-DP, across large sample sets, while providing complete exon and intron sequences that significantly reduce ambiguous results (20). Studies have demonstrated that, relative to SBT, NGS achieves markedly superior resolution and faster turnaround times. It can directly provide high-resolution genotyping results and resolve ambiguous allele combinations that occur with conventional Sanger sequencing (21). Currently, widely used platforms such as Illumina Miseq and HiSeq combine the advantages of high throughput with relatively low cost, generating hundreds of gigabases of data per run and enabling large-scale HLA typing (22). Third-generation sequencing technologies, including PacBio’s single-molecule real-time (SMRT) sequencing and Oxford Nanopore’s MinION platform, provide much longer read lengths capable of spanning entire HLA genes, thereby further eliminating phasing ambiguities (23, 24). With the continued decline in sequencing costs, NGS-based typing is becoming increasingly accessible and is expected to emerge as the new standard for HLA genotyping. A comparative analysis of the resolution, throughput, cost, and core application scenarios of these genetic testing technologies is presented in **Table 2**.

**Table 2:** Comparative analysis of genetic testing technologies.

Technology Type	resolution	Speed and Flux	cost	Information content and added value	Core application scenarios
PCR-SSP/SSO (13–15)	Low (gene/antigen level)	Rapid requires several hours; low throughput with limited sites per run.	Low cost, inexpensive reagents, simple equipment	Only the basic classification is obtained, with no additional information.	Routine HLA Typing Screening
Sequencing-Based Typing (16–18)	Medium-high (at the allele level, confined to target regions)	Slower, requiring 1-2 days per sample, with low throughput.	Mid-to-high range, several hundred dollars per specimen	Obtaining allele sequences can resolve ambiguous results, but it is difficult to discover new loci.	Classification and confirmation of high-risk cases
NGS genotyping (19–24)	Ultra-high coverage (whole genome sequencing, including exons and introns)	Relatively slow, requiring several days to process; High throughput, handling tens to hundreds of samples per batch.	Moderate, with high equipment investment, and decreasing per-sample costs.	Obtained complete sequences, identified novel alleles, resolved 76%–91% of Sanger sequencing ambiguities, and supported synchronous genotyping across multiple loci.	Large-scale high-resolution donor-recipient matching
Non-HLA genetic testing (37–49)	Moderate (KIR: present/absent; cytokine: SNPs)	Moderate, PCR sequencing reactions require several days; moderate throughput	Moderate, based on the number of detection sites	Aids in the assessment of immunocompatibility by providing supplementary information beyond HLA	Scientific research and exploration, High-risk case assessment support
dd-cfDNA testing (76–88)	High (quantitative proportion, reflecting the severity of damage)	Moderate, dPCR within hours, NGS within days; medium-to-high throughput	Moderate to high, commercial testing is costly, less expensive than biopsy.	Non-invasive assessment of graft injury, early warning of rejection, dynamic monitoring of therapeutic efficacy	Postoperative rejection non-invasive monitoring
Single-cell sequencing/Multi-omics (97, 98)	Extremely high (single-cell gene expression, multidimensional molecular profiles)	Slow, complex procedures, time-consuming data analysis; low throughput	High, with substantial equipment and analytical costs	Analyzing the immune microenvironment, discovering novel mechanisms, and constructing predictive models holds significant scientific value.	Fundamental research, mechanism exploration
Epigenetic testing (99–102)	High (quantitative methylation levels, non-coding RNA levels)	Moderate, PCR sequencing requires several days; moderate throughput	High, with demanding technical requirements	Assessing immune tolerance and predicting infection risk from an epigenetic regulation perspective	Scientific research and exploration, identification of tolerant populations

PCR-SSP, Polymerase Chain Reaction with Sequence-Specific Primers; NGS, Next-Generation Sequencing; dd-cfDNA, Donor-derived cell-free DNA; HLA, Human Leukocyte Antigen; KIR, Killer Immunoglobulin-like Receptor.

#### 2.1.2 Clinical impact: the survival benefit of high-resolution matching

Transplant immune rejection is primarily driven by donor–recipient HLA disparities, making accurate HLA typing critical for improving transplant success rates (25). Traditional low-resolution typing methods provide only antigen-level matching, for example HLA-A2 or HLA-B15, and fail to capture the extensive allelic polymorphisms that underlie immune responses. This limitation often leads to underestimation of potential immunological risk (18). By contrast, high-resolution HLA typing using NGS enables precise allele-level matching, such as distinguishing HLA-A02:01 from HLA-A02:06, across all class I loci (HLA-A, B, C) and class II loci (HLA-DR, DQ, DP) (26). This approach significantly reduces the risk of both acute rejection and chronic allograft injury.

Clinical evidence indicates that complete HLA allele matching confers a significant survival benefit. Data from large-scale analyses, such as the CTS study, show that kidney transplants with full HLA compatibility achieve markedly superior long-term outcomes: the 10-year graft survival rate was about 55% in HLA-identical transplants versus 38-40% with 5–6 mismatches, and the median graft half-life was prolonged from 8.5–9 years to 12.5 years under complete compatibility (27).

HLA-DQ and HLA-DP loci, often overlooked in traditional typing due to their complex polymorphisms, have now been recognized as independent risk factors for rejection. A retrospective cohort study of 788 kidney transplant recipients found that patients with one or two HLA-DQ mismatches had a significantly higher risk of rejection compared with those with no mismatches. Late rejection was particularly increased, and the presence of DQ mismatches was closely associated with antibody-mediated rejection (ABMR) (28). Furthermore, combined mismatches at HLA-DQ and HLA-DR loci synergistically amplified the risk of acute rejection, suggesting an additive immunogenic effect. Similarly, a meta-analysis of five studies including 1,166 kidney transplant recipients demonstrated that the development of *de novo* donor-specific antibodies (DSAs) against HLA-DP significantly increased the risk of graft loss and acute rejection (29). In addition, a retrospective case-control study showed that the presence of preformed isolated HLA-DP DSAs was an independent predictor of post-transplant ABMR, with affected patients exhibiting higher microvascular inflammation scores and more severe transplant glomerulopathy on biopsy (30).

For broadly sensitized patients, defined by a panel-reactive antibody level greater than 80%, high-resolution HLA typing allows detailed characterization of anti-HLA antibody specificity. In combination with donor typing, this facilitates the establishment of an “acceptable mismatch” list, thereby increasing the likelihood of identifying compatible donors. Before desensitization therapies such as plasmapheresis or immunoadsorption, determining whether recipient antibodies target specific donor HLA alleles helps predict treatment success and avoid high-risk donors. Moreover, in the context of marginal donor kidneys, such as those from older donors or those with comorbidities, selecting recipients with minimal HLA mismatches based on high-resolution typing can partly offset donor-related limitations and improve graft prognosis.

### 2.2 Novel molecular-level strategies for HLA matching

The traditional metric of “allele mismatch count” does not accurately capture the true immunogenicity of HLA molecules. In contrast, novel molecular-level assessment tools based on structural and functional analysis now allow quantification of immunological risk, thereby enhancing the precision of donor–recipient matching.

#### 2.2.1 Epitope mismatch analysis

Epitopes, defined as clusters of three to five spatially adjacent amino acids on the surface of HLA molecules, represent the fundamental units recognized by antibodies and T cells. When donor and recipient HLA molecules differ at these critical epitopes, an “epitope mismatch” occurs, which may trigger alloimmune responses (31). Computational algorithms such as HLAMatchmaker can analyze high-resolution donor-recipient HLA typing data to predict and quantify the number and location of epitope mismatches (32). Compared with conventional antigen-level mismatch assessment, epitope mismatch analysis provides a more precise measure of immunogenicity. A large multicenter study involving 5159 kidney transplant recipients demonstrated a strong correlation between the number of epitope mismatches in class II HLA molecules, particularly HLA-DQ and HLA-DR, and the risk of ABMR. The greater the number of mismatched epitopes, the higher the likelihood of ABMR, thereby offering a more refined tool to identify patients whose immunological risk may be underestimated by traditional matching methods (33).

#### 2.2.2 PIRCHE-II scoring

The Predicted Indirectly ReCognizable HLA Epitopes (PIRCHE-II) algorithm focuses on the indirect pathway of allorecognition, in which donor HLA molecules are degraded into peptides (epitopes) that are subsequently presented by the recipient’s class II HLA molecules to recipient T cells, thereby activating alloimmune responses (34). By analyzing the amino acid sequence of donor HLA molecules, this algorithm predicts which peptides can be presented by the recipient’s class II HLA molecules and calculates the total number of indirectly recognizable epitopes, expressed as the PIRCHE-II score. A high PIRCHE-II score indicates that donor HLA molecules harbor a large number of epitopes capable of being indirectly recognized by the recipient’s immune system, implying an elevated immunological risk. The aforementioned large multicenter study also confirmed its clinical utility, demonstrating that PIRCHE-II scores derived from HLA-DQB1 and DRB1 were significantly associated with the risk of ABMR. These findings suggest that PIRCHE-II scoring may provide valuable guidance for tailoring individualized immunosuppressive regimens (33).

#### 2.2.3 HLA evolutionary divergence

HLA evolutionary divergence (HED) quantifies the degree of difference between donor and recipient HLA molecules by calculating their evolutionary distance on a phylogenetic tree (35). In theory, greater evolutionary distance reflects larger structural and functional disparities between HLA molecules, which in turn may confer stronger immunogenicity. HED is computed using the amino acid sequences of HLA alleles, with tools such as midasHLA enabling its estimation. However, the clinical utility of HED in kidney transplantation remains controversial. While some studies in liver transplantation have reported associations between HED and rejection risk (36), large-scale cohort studies in kidney transplantation have not confirmed an independent link between HED and ABMR (33). As a result, routine application of HED in kidney transplant matching is not currently recommended.

### 2.3 Non-HLA immunological risk assessment

In addition to HLA alleles, non-HLA genetic factors can influence transplant immune responses by modulating pathways such as innate immunity and inflammatory processes. Although their effects are generally weaker than those of HLA, they provide an important supplementary dimension for evaluating donor–recipient immunological compatibility. At present, most non-HLA genetic testing remains in the research stage, and its clinical significance requires further validation.

#### 2.3.1 KIR genotyping and ligand matching

The killer immunoglobulin-like receptor (KIR) gene family encodes receptors on natural killer (NK) cells, with their primary ligands being class I molecules such as HLA-C. The recipient’s KIR genotype, in combination with the donor’s HLA ligands, is thought to influence NK cell-mediated immune responses to the graft (37). When a recipient carries certain activating KIRs but the donor lacks the corresponding HLA ligand, a scenario referred to as KIR-ligand mismatch, NK cells may be more readily activated and thereby contribute to graft rejection. Several studies have suggested that incorporating KIR/HLA compatibility into donor-recipient evaluation may improve transplant outcomes, although findings have not been entirely consistent (38, 39). Currently, KIR genotyping is performed mainly through PCR and sequencing to determine the presence or absence of specific KIR genes, providing an auxiliary tool for assessing non-HLA immunological compatibility. As understanding of the role of innate immunity in transplantation deepens, KIR-ligand matching may emerge as a relevant consideration in donor allocation for high-risk recipients.

#### 2.3.2 Cytokine gene polymorphisms

The intensity of the recipient’s immune response is partly regulated by cytokine gene polymorphisms. Cytokines such as IL-2, IL-10, TNF-α, TGF-β, and IFN-γ play important regulatory roles in transplant immunology, and single nucleotide polymorphisms (SNPs) within their genes can alter cytokine expression levels, thereby influencing the balance between graft tolerance and rejection (40). For the IL-2 gene, the promoter region -330 T/G polymorphism has been associated with reduced secretion in carriers of the T allele, and homozygosity has been reported to increase the risk of acute rejection (AR) (41). However, meta-analyses have failed to confirm a significant association, suggesting that the influence of IL-2 polymorphisms on AR remains inconclusive. In the IL-10 gene, several promoter polymorphisms (including -1082 G/A, -819 C/T, and -592 C/A) theoretically modulate anti-inflammatory activity and could influence rejection risk. Nevertheless, large-scale meta-analyses have not demonstrated significant correlations, indicating only a limited contribution of IL-10 polymorphisms to AR (42).

In contrast, the TNF-α gene -308 G/A polymorphism has shown more consistent results. The A allele (TNF2), associated with high TNF-α expression, has been repeatedly linked to an increased risk of AR, with pooled odds ratios around 1.4, particularly in severe and steroid-resistant cases, and is considered a major genetic susceptibility factor for AR (43). If the donor carries proinflammatory genotypes, such as high-expression variants of TNF-α, the intrinsic cells of the allograft may secrete increased levels of chemokines after transplantation, thereby exacerbating immune cell infiltration. By contrast, the IFN-γ +874 A/T polymorphism has emerged as a robust marker, with the T allele linked to high cytokine production and consistently associated with an increased risk of AR, particularly in Caucasian populations and in deceased-donor kidney transplant recipients (44). This variant is now regarded as a strong genetic predictor of AR. Additional cytokine-related genes, such as IL-6 and its receptor polymorphisms, have also been implicated in AR risk (45), further expanding our understanding of the contribution of cytokine pathway genetic variation to transplant outcomes. Overall, cytokine gene polymorphism testing holds promise for identifying individuals at high risk of AR and guiding personalized immunosuppressive therapy.

#### 2.3.3 Non-HLA antigen gene testing targets

Certain non-HLA antigens, such as MHC class I chain-related gene A (MICA) and the angiotensin II type 1 receptor (AT1R), can induce the formation of alloantibodies in transplant recipients, and these antibodies have been suspected to contribute to graft rejection (46). Laboratory testing for anti-MICA or anti-AT1R antibodies can be performed using ELISA or flow cytometry, both before and after transplantation. Some studies have reported that patients with anti-MICA antibodies exhibit significantly lower one-year graft survival compared with those without such antibodies, 88% versus 93%, suggesting that anti-MICA antibodies may impair graft function (47). Other investigations have indicated an association between anti-AT1R antibodies, rejection episodes, and graft dysfunction (48, 49). However, conflicting evidence also exists, with some studies failing to demonstrate significant adverse effects or statistical correlations. As a result, the clinical significance and interpretation of non-HLA antigen gene testing remain controversial, and these assays have not yet been incorporated into routine evaluation protocols.

### 2.4 Genotypic risk stratification: donor quality and host susceptibility

#### 2.4.1 Donor quality assessment: APOL1

Although the Apolipoprotein L1 (APOL1) gene does not directly affect drug metabolism, its variants are strongly associated with long-term graft outcomes, particularly in individuals of African ancestry. Carriers of high-risk APOL1 alleles, specifically the G1 or G2 variants, are more prevalent in this population and have been linked to an increased incidence of primary kidney diseases such as focal segmental glomerulosclerosis (FSGS). When donor kidneys are derived from individuals homozygous for high-risk alleles (G1/G1 or G2/G2), the risk of late allograft failure is nearly doubled compared with grafts from donors without these variants (50). Emerging evidence also suggests that African American recipients carrying high-risk APOL1 alleles may experience higher rates of T cell–mediated rejection and graft loss. This is hypothesized to be related to APOL1 expression and regulatory roles in immune cells, although further studies are needed for validation. Incorporating APOL1 genotyping into donor evaluation provides valuable prognostic information and may help predict long-term outcomes and guide recipient selection (51, 52).

#### 2.4.2 Host infection susceptibility genes

Polymorphisms in innate immune genes significantly influence individual susceptibility to infection and the strength of the immune response. By detecting these genetic variants, it is possible to identify transplant recipients at elevated risk of infection and to implement early adjustments to immunosuppressive regimens and preventive strategies. Among the most critical markers is Toll-like receptor 4 (TLR4), a key receptor responsible for recognizing lipopolysaccharides from Gram-negative bacteria. A common polymorphism, Asp299Gly (rs4986790), reduces receptor functionality, thereby weakening the inflammatory response to endotoxin stimulation. This presents a double-edged effect: on one hand, TLR4 polymorphisms attenuate recipient responsiveness to alloantigen stimulation, with one study reporting nearly a 60% reduction in acute rejection rates (53). On the other hand, this compromised innate immune defense significantly increases the risk of severe infection. In the same study, carriers of the TLR4 299Gly polymorphism demonstrated a markedly higher incidence of severe bacterial infections and a threefold increase in opportunistic infections. Furthermore, patients carrying both Asp299Gly and Thr399Ile polymorphisms exhibit a significantly elevated risk of CMV-related disease (54).

Beyond TLR4, other genetic factors also contribute to infection risk. Nucleotide-binding oligomerization domain-containing protein 2 (NOD2) is an intracellular pattern recognition receptor involved in bacterial sensing, and its polymorphisms have been associated with an increased risk of postoperative intra-abdominal infections in kidney transplant recipients (55). Similarly, the DEFB gene encodes defensins, a family of antimicrobial peptides; variants in this gene influence defensin expression levels and have been linked to increased susceptibility to fungal infections such as candidiasis (56).

Consequently, preoperative risk stratification is essential. Screening for recipient susceptibility genes such as TLR4, NOD2, and DEFB, in combination with donor infection-related indicators like CMV serostatus, enables the classification of recipients into low-, intermediate-, or high-risk categories for infection. This stratification provides an evidence-based framework for tailoring postoperative prevention and management strategies (57).

## 3 Peri-operative: pharmacogenomics-guided initiation of immunosuppression

Kidney transplant recipients require lifelong use of immunosuppressive agents, which are characterized by a narrow therapeutic window and marked interindividual variability in response. The primary determinant of this variability lies in genetic polymorphisms of drug-metabolizing enzymes, transporters, and pharmacological targets. Pharmacogenomics enables the analysis of these genetic features to predict a patient’s metabolic capacity and drug responsiveness, thereby guiding personalized dosing strategies that balance efficacy with toxicity, while reducing the risk of both rejection and adverse drug reactions.

### 3.1 CYP3A5 and dose optimization of tacrolimus

Tacrolimus is a frontline immunosuppressant in kidney transplantation and is primarily metabolized by the CYP3A enzyme system, with CYP3A5 serving as the major metabolic enzyme. A SNP in intron 3 of the CYP3A5 gene (rs776746) defines two principal alleles: the functional *1 allele associated with active enzyme expression, and the loss-of-function *3 allele associated with no active enzyme expression (58). Consequently, individuals carrying at least one *1 allele (genotypes *1/*1 or *1/*3, representing approximately 10%-30% of the population) are classified as “extensive metabolizers,” characterized by normal CYP3A5 activity and higher tacrolimus clearance. In contrast, individuals with the *3/*3 genotype are considered “poor metabolizers” or “non-expressers,” lacking CYP3A5 activity, which results in slower drug clearance and an increased risk of drug accumulation (59).

In clinical practice, these genetic distinctions have significant therapeutic implications. CYP3A5 extensive metabolizers exhibit lower tacrolimus trough concentrations following oral administration and therefore require an initial dose 1.5- to 2-fold higher than that prescribed for poor metabolizers to achieve therapeutic targets (60). Conversely, poor metabolizers given standard doses are prone to drug accumulation, which increases the risk of nephrotoxicity and neurotoxicity.

This genotype-guided approach is supported by robust evidence. Randomized controlled trials (RCTs) comparing “CYP3A5 genotype-guided dosing” with “conventional weight-based dosing” demonstrated that the genotype-guided group achieved target tacrolimus concentrations significantly earlier (61). Specifically, this approach reduced the time to reach the therapeutic range by approximately 50% and resulted in a 30% reduction in intrapatient variability of drug exposure. Studies in Chinese kidney transplant recipients further confirmed that extensive metabolizers required substantially higher average daily doses to achieve target concentrations compared with poor metabolizers (62, 63). Accordingly, both the Clinical Pharmacogenetics Implementation Consortium (CPIC) and the European Renal Best Practice (ERBP) guidelines now recommend pre-transplant CYP3A5 genotyping. For extensive metabolizers, an initial tacrolimus dose of 0.15-0.20 mg/kg/day is advised, whereas for poor metabolizers, a lower starting dose of 0.10-0.12 mg/kg/day is recommended (64). Several transplant centers have already incorporated this testing into routine preoperative assessments to optimize immunosuppressive therapy.

### 3.2 Combined influence of CYP3A4 and ABCB1

CYP3A4 serves as a secondary metabolic enzyme for tacrolimus, and its *22 allele (rs2161722) has been associated with reduced enzymatic activity, leading to decreased clearance and elevated blood concentrations of tacrolimus (65). Although the effect of CYP3A4 is less pronounced than that of CYP3A5, in CYP3A5 non-expressers (*3/3 genotype), CYP3A422 may represent the predominant determinant of tacrolimus metabolism, warranting careful dose adjustment (66). The ABCB1 gene encodes P-glycoprotein (P-gp), a key efflux transporter expressed in the intestine, liver, and kidney, which modulates tacrolimus absorption and systemic distribution (67). Polymorphisms such as C3435T (rs1045642) have been reported to influence P-gp expression and function, thereby indirectly affecting tacrolimus concentrations (68). Considering the combined genotypes of CYP3A5, CYP3A4, and ABCB1 provides a more comprehensive prediction of tacrolimus metabolic phenotypes and enables further optimization of dosing strategies (69).

### 3.3 UGT1A9 and dose adjustment of mycophenolic acid

Mycophenolate mofetil (MMF) and enteric-coated mycophenolate sodium (EC-MPS) are prodrugs whose active metabolite, mycophenolic acid (MPA), is primarily metabolized by uridine diphosphate glucuronosyltransferase 1A9 (UGT1A9) into the inactive compound mycophenolic acid glucuronide (MPAG). Consequently, polymorphisms in the UGT1A9 gene have a significant impact on MPA clearance (70). Specifically, promoter variants such as -275T>A and -2152C>T increase the transcriptional activity of the enzyme, accelerating MPA glucuronidation and raising clearance by 40%-60%, which reduces the area under the curve (AUC) by approximately 30% (71). Patients with this “fast metabolizer” profile require a 20%–30% higher MMF dose to maintain therapeutic exposure, as insufficient immunosuppression may otherwise elevate the risk of acute rejection.

Conversely, coding region variants such as *3 reduce enzymatic activity, leading to MPA accumulation and increasing the risk of adverse events, including bone marrow suppression and gastrointestinal toxicity. Clinical evidence supports these pharmacological distinctions; a study in stable kidney transplant recipients demonstrated that carriers of the UGT1A9 -275A allele had significantly lower dose-normalized MPA AUC values compared with non-carriers (72). Furthermore, evidence confirms that UGT1A9 rapid metabolizers, who typically exhibit subtherapeutic MPA levels, face a nearly two-fold greater risk of acute rejection compared to slow metabolizers, highlighting the importance of genotype-guided dosing to personalize therapy and mitigate this risk (73). Specific recommendations for dosage adjustment based on immunosuppressant-associated genetic polymorphisms are detailed in **Table 3**.

**Table 3:** Dose adjustment for immunosuppressant-associated genetic polymorphisms.

Immunosuppressant	Related gene polymorphisms	Genotype/Phenotype	Effects on drug metabolism	Dosage adjustment recommendations
Tacrolimus	*CYP3A5* (rs776746) (58–64)	Fast metabolism type (*1/*1, *1/*3)	High CYP3A5 enzyme expression results in rapid drug clearance and low blood concentrations.	The initial dose is recommended to be 1.5 to 2 times the standard dose (0.15-0.20 mg/kg/day), with close monitoring.
		Slow Metabolism Type (*3/*3)	CYP3A5 enzyme is not expressed, resulting in slow drug clearance.	Administer standard or lower initial doses (0.10-0.12 mg/kg/day), and remain vigilant for toxicity.
	*CYP3A4* (rs2161722) (65, 66)	A/A or A/G (*22 allele)	Reduced CYP3A4 enzyme activity, resulting in slower drug clearance	It may be necessary to reduce the dosage to avoid toxicity.
		G/G (wt)	CYP3A4 enzyme activity is normal	Standard dose
	*ABCB1* C3435T (rs1045642) (67–69)	T/T	P-gp expression or function may be reduced, resulting in increased bioavailability.	It may be necessary to reduce the dosage.
		C/C or C/T	P-gp function is normal	Standard dose
Cyclosporine	*CYP3A5* (rs776746) (58)	Fast metabolism type (*1/*1, *1/*3)	High expression of CYP3A5 enzyme results in rapid drug clearance.	It may be necessary to increase the dosage to achieve the target concentration.
		Slow Metabolism Type (*3/*3)	CYP3A5 enzyme is not expressed.	Standard dose
Mycophenolic Acid	*UGT1A9* -275T>A, -2152C>T (70–73)	Carrier of a variant allele	Enhanced UGT1A9 enzyme activity accelerates drug clearance, resulting in low blood concentrations.	It may be necessary to increase the dosage (20-30% higher) to achieve therapeutic effect.
		Wild-type	UGT1A9 enzyme activity is normal	Standard dose

### 3.4 Genotype-based dose calculation models

Genotype-based dosing models for immunosuppressants have been developed to improve the precision of pharmacotherapy in kidney transplantation. Single-gene approaches, such as CYP3A5-guided tacrolimus dosing, have been exemplified by a simplified formula in which the dose (mg/kg/day) is calculated as 0.12 + 0.08 × number of CYP3A51 alleles, allowing for rapid clinical decision-making. More advanced multigene models incorporate additional variants in CYP3A4, ABCB1, and UGT1A9, together with clinical covariates such as body weight, age, ethnicity, and hepatic function, to predict drug exposure more accurately (74). Compared with single-gene strategies, these integrated models have improved the accuracy of AUC prediction by approximately 20-30%, and are increasingly being implemented in large transplant centers, following the conceptual framework of pharmacogenomic models such as the International Warfarin Pharmacogenetics Consortium (IWPC) algorithm (75).

## 4 Post-transplantation: surveillance and long-term management

The post-transplantation phase shifts the clinical focus from acute surgical recovery to the long-term maintenance of graft health. This phase employs genetic testing for the early detection of rejection, dynamic monitoring of infection, and management of long-term drug toxicity.

### 4.1 Noninvasive monitoring of rejection

Acute rejection, particularly ABMR, is a major cause of kidney allograft failure. Traditional monitoring methods rely on serum creatinine, which is limited by delayed response and poor specificity, and renal biopsy, which is invasive and carries procedural risks. Advances in genetic testing have established a dual-dimension, blood- and urine-based noninvasive monitoring system that enables early warning, precise differentiation, and therapeutic response assessment for rejection episodes.

#### 4.1.1 Donor-derived cell-free DNA testing

Donor-derived cell-free DNA (dd-cfDNA) represents a revolutionary breakthrough in transplant monitoring, serving as a highly sensitive “liquid biopsy.” Biologically, dd-cfDNA consists of short DNA fragments (typically 120 to 160 base pairs) released into the recipient’s circulation following the apoptosis or necrosis of donor organ cells. Under stable graft function, these levels remain extremely low due to their short half-life (76). However, during immune rejection or significant graft injury, extensive cell death triggers a marked release of dd-cfDNA (77). By utilizing highly sensitive sequencing or digital PCR technologies, the quantitative measurement of plasma dd-cfDNA provides a direct, noninvasive window into the status of the kidney allograft (78, 79).

Regarding diagnostic accuracy and clinical utility, dd-cfDNA has demonstrated superior performance compared to traditional markers. It is particularly effective in detecting ABMR, the leading cause of late graft failure. Multiple studies have confirmed its efficacy; for instance, a meta-analysis reported a pooled sensitivity of 0.81 and specificity of 0.80 for diagnosing ABMR, with an area under the receiver operating characteristic curve (AUC) of 0.87 (80). In prospective studies using a cutoff threshold of 1%, dd-cfDNA effectively identified rejection with high specificity (>90%) and proved capable of detecting even “non-DSA-associated ABMR” (81, 82). This precision significantly outperforms serum creatinine; ROC analysis has shown that dd-cfDNA achieves an AUC of 0.804 compared to 0.609 for creatinine, confirming its ability to capture subclinical injury that functional markers miss (83). While its sensitivity for T cell-mediated rejection (TCMR) is more moderate at approximately 70%, likely due to the localized nature of tubulointerstitial injury where DNA is readily phagocytosed, the aggregate clinical benefits are substantial (84). Randomized interventional trials have demonstrated that using dd-cfDNA to guide biopsy decisions shortened the time to ABMR diagnosis from 14.5 months to 2.8 months while reducing unnecessary invasive biopsies by 30% (85). Furthermore, its high negative predictive value enables clinicians to confidently rule out moderate-to-severe rejection when levels are low, and it serves as a dynamic marker for therapeutic monitoring, with effective treatment typically leading to a rapid decline in levels within 2 to 4 weeks (86).

However, the interpretation of dd-cfDNA requires caution, particularly regarding its specificity in the context of infection. Elevated dd-cfDNA levels are not exclusive to alloimmune rejection but can occur due to any process causing graft cell necrosis, including active infections. Observational studies indicate that dd-cfDNA levels are markedly elevated in BK virus-associated nephropathy (BKVN) and mixed rejection, in some instances even exceeding levels seen in isolated ABMR (87). Therefore, to ensure diagnostic accuracy and avoid misinterpreting infection-related injury as rejection, dd-cfDNA results should always be integrated with clinical parameters, such as viral load monitoring. In contrast, levels typically remain significantly lower in cases of calcineurin inhibitor (CNI) toxicity, recurrent glomerulonephritis, and histologically normal grafts, aiding in the exclusion of these conditions (88).

#### 4.1.2 Gene expression profile monitoring

Changes in the expression of immune-related genes in the recipient’s peripheral blood or urine can serve as molecular signatures of rejection, reflecting systemic or local immune activation. At the systemic level, measuring the mRNA expression levels of immune-related genes in peripheral blood allows for the calculation of a ‘rejection risk score’ to assess immune activity. For example, the kSORT assay analyzes the expression of over 20 immune-related genes and achieves a negative predictive value (NPV) of 98% for immune quiescence, thereby reducing the need for protocol biopsies in low-risk patients (89). Similarly, TruGraf employs microfluidic PCR to detect differentially expressed genes in blood, enabling the identification of biopsy-proven subclinical rejection despite normal clinical function. With an AUC of 0.82, it is considered suitable for routine surveillance (90).

Complementing blood analysis, urine originates directly from the transplanted kidney and thus serves as an ideal indicator for monitoring the local immune status of the graft. Notable biomarkers include CXCL9 and CXCL10, which are T-cell chemoattractants. During TCMR, increased renal T-cell infiltration leads to the significant release of these chemokines into the urine (91, 92). Multicenter validation studies have demonstrated a negative predictive value exceeding 99%, allowing progressive rejection to be excluded before renal dysfunction becomes apparent (93). Consequently, in the 2022 European Society for Organ Transplantation (ESOT) consensus, experts unanimously recommended urinary CXCL9/10 as clinical monitoring markers for acute rejection (94). Additionally, the co-inhibitory receptor TIM-3 expressed on effector cells has shown prognostic value (95); sequential monitoring in cohort studies demonstrated that urinary TIM-3 mRNA expression at 3 and 6 months post-transplant effectively distinguished patients who subsequently developed allograft dysfunction from those with stable graft function, showing significant differences between the two groups (96).

#### 4.1.3 Emerging technologies: multi-omics and epigenetics

Recent breakthroughs in biomedical science have given rise to a range of emerging genetic testing technologies for kidney transplant monitoring, including single-cell analysis and epigenetic profiling. Single-cell RNA sequencing (scRNA-seq) and related approaches enable the characterization of gene expression profiles and clonal information at the resolution of individual immune cells within graft tissue or peripheral blood (97). In the transplantation field, these techniques have been applied to renal allograft biopsy samples to precisely identify the cellular composition of infiltrates, such as distinct T-cell subsets and activation states of macrophages. These findings have provided novel perspectives on the mechanisms underlying ABMR and other rejection processes (98).

In parallel, transplant immune regulation is also significantly influenced by epigenetic mechanisms such as DNA methylation, histone modifications, and noncoding RNAs. For example, the function of regulatory T cells (Tregs) is closely associated with the methylation status of the FOXP3 gene promoter (99). Studies have shown that kidney transplant recipients who achieve operational tolerance exhibit a significantly higher proportion of memory Tregs and greater demethylation of the FOXP3 TSDR region compared with patients who rely on immunosuppressive therapy (100). Additionally, small noncoding RNAs such as microRNAs (miRNAs) are of considerable interest; upregulation of urinary or circulating miRNAs such as miR-99a and miR-155 has been correlated with acute rejection, suggesting that detecting these miRNA signatures may serve as a useful adjunct for noninvasive diagnosis (101, 102). The testing parameters, advantages, and clinical applications of these emerging non-invasive monitoring technologies are outlined in **Table 4**.

**Table 4:** Emerging non-invasive monitoring technologies.

Technical Name	Testing parameters	Advantages	Clinical application
dd-cfDNA (76–88)	Proportion of donor-derived cell-free DNA	High sensitivity, non-invasive, early warning	Rejection reaction monitoring, efficacy assessment
Peripheral blood mRNA (89, 90)	Dynamic monitoring of peripheral blood gene expression	Early identification of abnormal immune activation states	Assessing systemic immune activity status, ruling out subclinical rejection
scRNA-seq (97, 98)	Single-cell immunome	High resolution, revealing the immune microenvironment	Research into rejection mechanisms
Epigenetic markers (99–102)	DNA methylation (FOXP3), microRNA	Reflecting the functional state of immune cells	Assessing immunotolerance status
Urinary mRNA (91–96)	Expression of CXCL9/CXCL10 and Tim3 in Urine	Consistent with renal T-cell infiltration, strong negative predictive value	Non-invasive diagnosis to predict the risk of rejection/complications
Ex vivo perfusion expression profiling (113)	Rapid gene expression detection using infusion solution	Optimizing the efficiency of kidney allocation	Assessing Organ Quality/IRI Burden intraoperatively

scRNA-seq, Single-cell RNA sequencing; CXCL9/10, Chemokine (C-X-C motif) ligand 9/10; Tim-3, T-cell immunoglobulin and mucin-domain containing-3; IRI, Ischemia-Reperfusion Injury.

### 4.2 Dynamic infection management

Kidney transplant recipients are highly susceptible to infections due to long-term immunosuppressive therapy, with a postoperative infection incidence exceeding 40% (103). Advances in genetic testing now provide a dual approach to infection management by integrating pathogen nucleic acid monitoring with host susceptibility gene assessment performed pre-transplantation.

#### 4.2.1 Pathogen nucleic acid monitoring

Pathogen nucleic acid detection, primarily using real-time quantitative PCR, allows for the monitoring of viral load and the identification of pathogen reactivation before the onset of clinical symptoms. For Cytomegalovirus (CMV), the most common opportunistic pathogen following kidney transplantation, regular monitoring of DNA load in recipient blood enables early detection of viral reactivation (104). Initiating preemptive antiviral therapy, such as valganciclovir, when the viral load exceeds 1000 copies/mL has been shown to reduce the incidence of CMV-related disease from 30% to 8% (105). Furthermore, dynamic monitoring during therapy facilitates the real-time assessment of treatment efficacy.

In the case of BK Virus (BKV), which can lead to BK virus-associated nephropathy (BKVN), a major cause of graft dysfunction, real-time quantitative PCR monitoring in urine and plasma is essential (106). Viral activation is typically indicated when the urinary load exceeds 10^7^ copies/mL or the plasma load exceeds 10^4^ copies/mL. Detection at these thresholds necessitates the timely adjustment of immunosuppressive regimens, such as reducing calcineurin inhibitor dosage or switching to less nephrotoxic agents (107). Implementing these interventions can reduce the incidence of BKVN from 15% to 5% (108).

#### 4.2.2 Stratified intervention strategies

Gene-based infection management requires the integration of pathogen load and host susceptibility genes to formulate individualized prevention and control strategies. Consequently, postoperative monitoring and intervention should be stratified according to the risk profile established pre-transplantation. In high-risk populations, such as recipients carrying TLR4 variants, viral load monitoring for CMV and BKV is recommended every two weeks during the first six months post-transplantation. For these patients, prophylactic antibiotic therapy should be extended to six months, and immunosuppressive regimens associated with lower infection risk, such as belatacept-based protocols rather than CNIs, should be prioritized. In contrast, for low-risk populations, a less intensive protocol is sufficient, comprising monthly viral load monitoring and prophylactic antibiotics for the first three months, alongside standard immunosuppressive regimens (109).

Furthermore, precision therapeutic adjustment is critical when infections arise. When pathogen loads are found to be elevated, NGS-based resistance gene testing should be utilized to guide the selection of sensitive antimicrobial agents. Particularly in high-risk populations, the intensity of immunosuppressive therapy should be moderately reduced during the course of infection treatment to prevent excessive immunosuppression that could facilitate pathogen dissemination.

### 4.3 Long-term drug optimization: SLCO1B1 and statins

Kidney transplant recipients have a high prevalence of hyperlipidemia, affecting more than 60% of patients, largely due to long-term use of glucocorticoids and CNIs. Statins are widely prescribed in this population to reduce cardiovascular risk, yet genetic polymorphisms in SLCO1B1 have been closely linked to the risk of statin-induced myotoxicity (110). Mechanistically, the SLCO1B1 gene encodes the hepatic transporter organic anion transporting polypeptide 1B1 (OATP1B1), which facilitates hepatic uptake and clearance of statins from the circulation. The SLCO1B1 *5 allele (rs4149056 C variant) reduces OATP1B1 transport activity, resulting in higher systemic exposure to statins and a significantly increased risk of myotoxicity, including myalgia and rhabdomyolysis.

Clinical studies have shown that carriers of the *5 allele (genotypes TC or CC) treated with standard doses of simvastatin exhibit a 2.2- to 2.6-fold higher risk of developing myotoxicity compared with non-carriers (111). Consequently, in clinical practice, patients identified as carrying the SLCO1B1 *5 allele are best managed either by selecting statins with minimal dependence on OATP1B1-mediated transport, exemplified by pravastatin, or by initiating therapy with a reduced statin dose, such as lowering simvastatin from 20 mg/day to 10 mg/day, to mitigate the risk of adverse drug reactions (112).

## 5 Cross-process considerations: ethics, implementation and future perspectives

### 5.1 Challenges and controversies in genetic testing

Genetic testing technologies hold great promise in kidney transplantation, yet their clinical implementation faces multiple challenges related to technical standardization, data interpretation, ethical considerations, clinical translation, and healthcare equity. Addressing these issues requires coordinated efforts across medicine, ethics, and policy. First, the technical and data infrastructure remains insufficient. High-throughput assays such as NGS are costly, labor-intensive, and lack standardized protocols for data processing and interpretation, limiting their adoption in many centers. Moreover, large-scale sequencing generates a vast number of variants of uncertain significance, and the absence of authoritative algorithms for risk evaluation increases the risk of clinical misinterpretation. Simplifying technologies, establishing unified guidelines, and training multidisciplinary professionals are urgently needed. Second, the available evidence for clinical efficacy and translation remains limited. Numerous genetic biomarkers, especially those beyond the HLA field, have been identified primarily in small-scale studies, often yielding inconsistent findings and lacking rigorous prospective validation. Their ability to meaningfully improve long-term transplant outcomes has yet to be proven. As a result, most of these tests remain confined to research settings, and their value in routine clinical practice is uncertain. Large multicenter collaborative studies are essential to clarify their clinical utility and define appropriate indications. Third, ethical, equity, and data security concerns are prominent. Genetic testing involves sensitive donor and recipient information, which raises risks of discrimination, such as the exclusion of donors based on their APOL1 status, dilemmas related to incidental findings, and potential data breaches (51). Unequal distribution of resources may further exacerbate healthcare disparities, as advanced technologies are more accessible in developed regions and large centers. Strengthening ethical frameworks, informed consent processes, and data security measures, alongside policy support for broader access, will be critical to ensure equitable and responsible application.

### 5.2 Future perspectives

Despite the challenges, genetic testing is expected to continue driving kidney transplantation toward the paradigm of precision medicine. Looking ahead, the convergence of multidimensional genetics with emerging technologies holds promise for propelling transplant medicine into an era of true precision. An outlook on the advantages and potential applications of these future technologies is provided in **Table 5**.

**Table 5:** Future technology outlook.

Technical Direction	Advantages	Potential applications
Polygenic risk score	Integrating multiple genetic risk factors	Comprehensive Risk Assessment
Microfluidics and CRISPR Detection	Portable, rapid genetic testing	Bedside real-time monitoring
Organoids and Immune Simulation	*In vitro* simulation of donor-recipient immune interactions	Predictive exclusion risk, personalized validation
NanoPore sequencing	Real-time, long-read sequencing	Intraoperative HLA matching, dynamic transcriptome monitoring
International Shared Database	System development, standardization of indicator thresholds	Enhancing comparability and accessibility

#### 5.2.1 Construction and application of polygenic risk scores

The integration of multidimensional genetic information, encompassing HLA epitope mismatches, pharmacogenomic variants such as CYP3A5 and UGT1A9, infection susceptibility genes such as TLR4, and graft outcome–associated genes such as APOL1, enables the development of transplant-specific polygenic risk scores that support comprehensive risk stratification across the transplant continuum. Intraoperatively, rapid gene expression profiling of perfusate markers such as IL-8 and lactate can inform real-time adjustments to perfusion strategies, thereby maximizing graft resuscitation (113). Pre-transplant PRS can quantify the combined risks of rejection, drug toxicity, and infection, thereby supporting donor selection and optimization of immunosuppressive regimens.

#### 5.2.2 Point-of-care testing driven by microfluidics and CRISPR technologies

Microfluidic chip platforms and CRISPR-based technologies are driving genetic testing toward true point-of-care applications, reducing dependence on centralized laboratories. Microfluidic chips can integrate sample preparation and detection workflows; for example, TruGraf employs microfluidic qPCR to rapidly analyze blood-based gene expression profiles (114). In the future, microfluidic technologies may allow the simultaneous performance of HLA typing and pharmacogenomic testing on the same platform, streamlining pre-transplant evaluation and supporting personalized immunosuppressive strategies. In parallel, the rapid development of CRISPR diagnostic methods is noteworthy. By harnessing the high specificity of CRISPR/Cas systems, these assays can be engineered to detect defined DNA sequences such as resistance-associated viral mutations or high-risk genotypes with high accuracy. When combined with isothermal nucleic acid amplification and simple fluorescence readouts, CRISPR diagnostics can deliver results within minutes using portable devices.

#### 5.2.3 Organoids and immune simulation for pre-transplant personalized validation

Organoid technology provides a valuable platform for “*in vitro* testing” in kidney transplantation. By deriving kidney organoids from recipient-induced pluripotent stem cells (iPSCs) and introducing donor-specific HLA genes through gene editing, it is possible to create donor–recipient matching models that can be co-cultured with recipient immune cells to quantify rejection risk and support donor selection (115). Researchers have demonstrated that iPSC-derived kidney organoids effectively mimic human kidney development and cellular diversity, exhibiting a high degree of reproducibility (116). This long-term expandable human kidney ‘tubular organoid’ model reproduces the key structures and functions of the nephron *in vitro* (117).

#### 5.2.4 Nanopore sequencing for real-time dynamic monitoring

Nanopore sequencing technology, characterized by its portability, long-read capability, and real-time data generation, is increasingly recognized as a promising tool in transplantation medicine. Recent studies have demonstrated that the portable MinION sequencer can complete full donor-recipient HLA genotyping within 24 hours, enabling intraoperative compatibility reporting (118). In the future, during kidney transplantation procedures, a single drop of blood introduced into a handheld sequencer may suffice to identify undetected HLA mismatches or unexpected genetic incompatibilities within just a few hours. Moreover, nanopore sequencing can directly profile RNA, thereby allowing real-time transcriptomic analyses. For instance, sequencing of recipient peripheral blood leukocyte mRNA could provide immediate insights into dynamic changes in immune-related gene expression, offering valuable guidance for postoperative immunosuppressive adjustments.

#### 5.2.5 International collaboration to promote equity and standardization

The establishment of global transplant genomics databases and shared platforms is crucial for advancing the field. Integrating genetic testing results with clinical outcome data will generate powerful resources to refine genotype-phenotype associations, validate biomarkers, and optimize risk prediction models. Such evidence-based insights can inform international guidelines and foster harmonized standards for quality control and data interpretation, thereby facilitating the broad and comparable application of genetic testing across regions.

## 6 Conclusion

Genetic testing technologies are reshaping the kidney transplantation care paradigm through comprehensive integration across the entire clinical continuum. This review distinguishes itself from prior literature by systematically synthesizing these advancements not as isolated tools, but as interconnected components of a precision medicine framework. From pre-transplant high-resolution HLA typing and epitope mismatch analysis for optimal donor-recipient matching, to post-transplant noninvasive monitoring with dd-cfDNA and urinary molecular biomarkers for early rejection detection; from pharmacogenomic-guided individualized immunosuppressive dosing to dual host-pathogen-oriented infection risk management, genetic testing provides powerful tools to address the long-standing challenges of chronic rejection and drug toxicity. This integrated approach is driving the field decisively from “experience-driven” to “data-driven” clinical decision-making.

Despite persistent challenges related to standardization, ethical and privacy considerations, and disparities in resource allocation, advances such as microfluidic point-of-care testing, nanopore sequencing, and organoid-based modeling, coupled with the establishment of international databases and harmonized guidelines, are progressively addressing these limitations and paving the way for broader clinical translation. Looking ahead, kidney transplantation is poised to undergo three major shifts: from passive management of rejection to proactive risk prevention, from uniform immunosuppressive protocols to precision-tailored therapy, and from centralized laboratory diagnostics to real-time bedside monitoring. With ongoing translational advances and clinical validation, genetic testing is expected to become a standard tool for comprehensive peri-transplant management, ultimately extending graft longevity, enhancing quality of life for patients with end-stage renal disease, and ushering in a new era of precision and individualized transplant medicine.
